# Integrative Proteomic and Phosphoproteomic Profiling Reveals Molecular Mechanisms of Hypoxic Adaptation in Brandt’s Voles (*Lasiopodomys brandtii*) Brain Tissue

**DOI:** 10.3390/cells14070527

**Published:** 2025-04-01

**Authors:** Panqin Wang, Yongyan Liu, Yimeng Du, Yiwen Gao, Tian Shao, Weifeng Guo, Zhenlong Wang, Han Cheng

**Affiliations:** 1 School of Life Sciences, Zhengzhou University, Zhengzhou 450001, China; 2 School of Electrical and Information Engineering, Zhengzhou University, Zhengzhou 450001, China

**Keywords:** Brandt’s voles, brain, hypoxia, proteomic, phosphoproteomic

## Abstract

Rapid ascent to high altitudes by unacclimatized individuals significantly increases the risk of brain damage, given the brain’s heightened sensitivity to hypoxic conditions. Investigating hypoxia-tolerant animals can provide insights into adaptive mechanisms and guide prevention and treatment of hypoxic-ischemic brain injury. In this study, we exposed Brandt’s voles to simulated altitudes (100 m, 3000 m, 5000 m, and 7000 m) for 24 h and performed quantitative proteomic and phosphoproteomic analyses of brain tissue. A total of 3990 proteins and 9125 phosphorylation sites (phospho-sites) were quantified. Differentially expressed (DE) analysis revealed that while protein abundance changes were relatively modest, phosphorylation levels exhibited substantial alterations, suggesting that Brandt’s voles rapidly regulate protein structure and function through phosphorylation to maintain cellular homeostasis under acute hypoxia. Clustering analysis showed that most co-expressed proteins exhibited non-monotonic responses with increasing altitude, which were enriched in pathways related to cytokine secretion regulation and glutathione metabolism, contributing to reduced inflammation and oxidative stress. In contrast, most co-expressed phospho-sites showed monotonic changes, with phospho-proteins enriched in glycolysis and vascular smooth muscle contraction regulation. Kinase activity prediction identified nine hypoxia-responsive kinases, four of which belonging to the CAMK family. Immunoblot validated that the changes in CAMK2A activity were consistent with predictions, suggesting that CAMK may play a crucial role in hypoxic response. In conclusion, this work discovered that Brandt’s voles may cope with hypoxia through three key strategies: (1) vascular regulation to enhance cerebral blood flow, (2) glycolytic activation to increase energy production, and (3) activation of neuroprotective mechanisms.

## 1. Introduction

With the expansion of the transportation network, increasing numbers of people are entering high-altitude regions for various purposes, including work, tourism, and military operations. Currently, approximately 25 million people reside at elevations exceeding 3000 m [1]. One of the characteristics of high-altitude environments is low atmospheric pressure and hypoxia, where the absolute content of oxygen gradually decreases progressively with elevation. Rapid ascent without proper acclimatization can lead to physiological disruptions in respiratory, cardiovascular, and nervous systems [2,3], potentially resulting in life-threatening conditions such as pulmonary and cerebral edema [4,5]. Therefore, a comprehensive understanding of oxygen response mechanisms can provide valuable perspectives for both elucidating life’s adaptive evolution and developing therapeutic strategies for hypoxia-related diseases.

The brain, as an aerobic organ with minimal energy reserves, is particularly vulnerable to hypoxic damage [4]. Remarkably, some species exhibit exceptional hypoxia tolerance. The thirteen-lined ground squirrel, for instance, maintains normal mitochondrial respiration during hibernation through elevated sulfide:quinone oxidoreductase activity in its brain, which oxidizes hypoxia-induced sulfides [6]. Similarly, the naked mole-rat has evolved unique adaptations, including the ability to utilize fructose for anaerobic metabolism in brain tissue [7], and tissue-specific regulation of the Akt/mTOR pathway through phosphorylation [8]. Phosphorylation, an essential protein post-translational modification, plays a pivotal role in regulating protein function, signal transduction, and hypoxia-related pathways. It is reported that Ladakhi women living at high altitudes (3250 m) possess elevated phosphorylation levels of nitric oxide synthase at S615 and S1177, which enhances oxygen transport [9].

*Lasiopodomys brandtii* (Brandt’s vole) is a small rodent that inhabits grasslands across regions of northeastern China, central and eastern Mongolia, and Russian border regions (1000–1200 m elevation) [10]. The population encounters hypoxic conditions due to the congregation of numerous individuals and burrow sealing [11,12]. Our previous research has confirmed its remarkable hypoxia tolerance, surviving for over 6 h in 5.5% O_2_ [13], making it a valuable model for hypoxic adaptation research. While transcriptomic analyses have characterized hypoxic responses in the brain [14,15], heart [16], and skeletal muscle [17] of Brandt’s voles, these findings are constrained by the limited correlation between transcript and protein levels. Advances in mass spectrometry present an exciting opportunity for comprehensively characterizing the proteome dynamics, as demonstrated in studies of naked mole-rats under hypoxia [18]. However, phosphoproteomic characterization of hypoxia-tolerant rodents remains unexplored.

In this study, we hypothesized that Brandt’s voles may employ rapid and dynamic regulation of protein phosphorylation to maintain cellular homeostasis and activate neuroprotective mechanisms under acute hypoxic condition. To test this hypothesis, we conducted proteome and phosphoproteome quantification on Brandt’s vole brains following 24 h exposures to simulated altitudes of 100 m, 3000 m, 5000 m, and 7000 m. We aimed to: (1) characterize altitude-dependent expression patterns at protein and phosphorylation levels; (2) identify hypoxia-responsive kinases; and (3) elucidate key signaling pathways and molecular targets in early hypoxic adaptation. This study represents the first phosphoproteomic characterization of hypoxia-tolerant rodents, providing insights for hypoxic adaptation and early prevention of altitude-related illnesses.

## 2. Materials and Methods

### 2.1. Animal Sampling and Maintenance

All experimental protocols were reviewed and approved by the Institutional Animal Care and Use Committee of Zhengzhou University, Zhengzhou, Henan, China (Approval No. ZZUIRB2022-44) and conducted in accordance with the Guidelines for the Use of Experimental Animals (China) and relevant local regulations. The Brandt’s voles were purchased from the Chinese Academy of Agricultural Sciences (Beijing, China) and maintained in an institute vivarium under controlled conditions (20–24 °C, 35–50% relative humidity, 12:12 h light–dark cycle) at Zhengzhou University. Healthy adult voles (12–14 weeks old; body weight 40–50 g; both sexes included) were selected for the study. One week prior to experiments, littermates were randomly assigned to different treatment groups and individually housed in polyethylene cages, with ad libitum access to food and water throughout the study period.

### 2.2. Hypoxia Exposure Protocol

Twenty adult voles were allocated into four experimental groups (*n* = 5 per group) corresponding to simulated altitudes of 100 m (normoxic control), 3000 m, 5000 m, and 7000 m. Hypobaric hypoxia was induced using a DSF-II hypobaric chamber (Huaxin Cabin, Weifang, China). Due to equipment limitations (single-chamber system), groups were processed sequentially under standardized conditions: (1) each group underwent a 24 h continuous exposure period; (2) subsequent groups were introduced immediately after chamber re-equilibration to baseline conditions (100 m); (3) all exposures commenced at 08:00 (±15 min); (4) chamber environment mirrored vivarium conditions.

### 2.3. Brain Tissue Collection and Processing

Following hypoxia exposure, voles were euthanized, and whole brain tissues were immediately collected, rinsed in ice-cold PBS, flash-frozen in liquid nitrogen, and stored at −80 °C until further analysis.

### 2.4. Protein Extraction and Quantification

Brain samples were ground on liquid nitrogen into powder and lysed in 8 M urea (Sigma-Aldrich, Saint Louis, MO, USA) containing 1% protease (Merck Millipore, Darmstadt, Germany) and phosphatase inhibitor cocktail (Millipore), followed by sonication for three minutes on ice. After that, the remaining debris was removed by centrifugation at 12,000× *g* for 10 min at 4 °C; the supernatants were carefully transferred to new centrifuge tubes, and protein concentration was quantified by BCA assay (Beyotime, Shanghai, China).

### 2.5. Mass Spectrometry Sample Preparation

Protein samples were reduced with 5 mM dithiothreitol (DTT, Sigma-Aldrich) at 56 °C for 30 min and alkylated with 11 mM iodoacetamide (IAA, Sigma-Aldrich) in the dark at room temperature (RT) for 15 min. Following dilution with 200 mM triethylammonium bicarbonate (TEAB, Sigma-Aldrich) to achieve a urea concentration below 2 M, proteins were digested with sequencing-grade trypsin (Promega, Madison, WI, USA) using a two-step protocol: initial digestion at 1:50 enzyme-to-protein ratio (37 °C, overnight) followed by secondary digestion at 1:100 ratio (37 °C, 4 h). Digested peptides were acidified to pH 2–3 using 10% trifluoroacetic acid (TFA, Sigma-Aldrich).

For desalting, Sep Pak tC18 cartridges (Waters) were first activated with 1 mL of methanol and equilibrated with 1 mL of 0.1% TFA, repeated five times. Following sample loading, cartridges were desalted using 1 mL of 0.1% TFA, repeated three times. Finally, the peptides were eluted with 1 mL of 80% acetonitrile (ACN, ThermoFisher Scientific, Waltham, MA, USA), repeated twice. The eluates were vacuum freeze-dried and stored at −20 °C for further use.

Peptides were fractionated using a 300 Extend C18 column (5 μm particle size, 4.6 mm inner diameter, 250 mm length) under high-pH reverse-phase HPLC conditions at RT. Mobile phase A consisted of 98% H_2_O + 2% ACN, adjusted to pH 9 with ammonia water, while mobile phase B consisted of 2% H_2_O + 98% ACN, also adjusted to pH 9. The peptide fractionation gradient was set to 8%–32% ACN (pH 9) at a flow rate of 1 mL/min, separating 60 fractions over 60 min. The fractions were then pooled into 4 groups, vacuum freeze-dried, and subjected to subsequent LC-MS/MS analysis.

For phospho-peptide enrichment, immobilized metal affinity chromatography (IMAC) materials were pre-washed with enrichment buffer (50% ACN/6% TFA; 3×). Peptides were dissolved in enrichment buffer and incubated with IMAC materials under gentle agitation. IMAC materials were washed three times with enrichment buffer (50% ACN/6% TFA). Following sequential washes with 50% ACN/6% TFA and 30% ACN/0.1% TFA (3× each), phosphopeptides were eluted with 10% ammonium hydroxide, desalted using C18 ZipTips (Millipore), and lyophilized for LC-MS/MS (liquid chromatography–tandem mass spectrometry) analysis.

### 2.6. Mass Spectrometry Data Acquisition

Peptides were dissolved in 0.1% formic acid (Buffer A) and separated using an EASY-nLC 1000 ultra-performance liquid chromatography system. Buffer B consisted of 0.1% formic acid in 90% acetonitrile. The LC gradient was programmed as follows: 3–17% B from 0 to 86 min, 17–28% B from 86 to 110 min, 28–80% B from 110 to 115 min, and maintained at 80% B from 115 to 120 min, with a constant flow rate of 500 nL/min.

Following LC separation, peptides were ionized using a nanoelectrospray ionization (NSI) source and analyzed by a Q Exactive™ Plus mass spectrometer (ThermoFisher Scientific). The ion source voltage was set to 2.2 kV. Both precursor ions and their fragments were detected using a high-resolution Orbitrap analyzer. The mass spectrometer was operated in data-dependent acquisition (DDA) mode, with full MS scans (*m*/*z* 350–1800) acquired at a resolution of 70,000, followed by MS/MS scans of the top 10 most intense precursor ions at a resolution of 35,000. Higher-energy collisional dissociation (HCD) was performed with a normalized collision energy of 28%. To optimize mass spectrometry efficiency, the automatic gain control (AGC) target was set to 1 × 10^5^, the intensity threshold to 20,000 ions/s, the maximum injection time to 100 ms, and the dynamic exclusion duration to 15 s to prevent repeated sequencing of abundant precursor ions.

### 2.7. Database Search

Raw secondary MS data were processed used MaxQuant (v1.6.6.0). The target protein database was generated from the transcriptome database based on the sequencing of Brandt’s voles. The common contamination library was added to the database. Search parameters included trypsin/P digestion with up to 2 missed cleavages; precursor mass tolerances of 20 ppm (initial search) and 5 ppm (main search); fragment mass tolerance of 0.02 Da; fixed modification of cysteine carbamidomethylation; variable modifications of methionine oxidation, N-terminal acetylation, and serine/threonine/tyrosine phosphorylation. Protein and peptide-spectrum match (PSM) false discovery rates (FDR) were set at 1%.

### 2.8. Data Processing

Label-free quantification (LFQ) intensity data were pre-processed using Perseus software (v1.6.14.0) [19]. Contaminants, reverse hits, and proteins with localization probability <0.75 were filtered out. Proteins and phospho-sites were retained for analysis if detected in at least three replicates within any experimental group. Then, data were Log_2_ transformed and median-scaled. Differential expression (DE) between hypoxic conditions and normoxic control was assessed using *t*-tests through the LIMMA package [20], which applies empirical Bayes shrinkage to standard errors and variance estimates for improved robustness in small-sample comparisons. The Benjamini–Hochberg method was used for multiple testing correction.

### 2.9. Functional Enrichment Analyses

Pathway annotation for Brandt’s vole proteins was performed using EggNOG-mapper (https://web.archive.org/web/20240324072322/http://eggnog-mapper.embl.de/, accessed on 1 March 2023) [21] and Reactome (https://reactome.org/, accessed on 1 March 2023) [22], generating three background sets: GO (Gene Ontology), KEGG (Kyoto Encyclopedia of Genes and Genomes), and Reactome. Subsequently, enrichment analysis of DE proteins and phospho-proteins was conducted using the enrich function in the clusterProfiler R package [23]. Enriched pathways with a *p*-value < 0.05 or lower were considered statistically significant.

Additionally, candidate genes involved in hypoxic adaptation were retrieved from the iHypoxia database (https://ihypoxia.omicsbio.info, accessed on 1 March 2023) [24] to investigate whether the DE proteins in Brandt’s voles have also been detected in other hypoxia-tolerant species.

### 2.10. Phospho-Site Function Scoring and Kinase Activity Prediction

Reference proteomes for humans and mice were downloaded from UniProt (https://www.uniprot.org/, accessed on 1 May 2023). Orthologous proteins of Brandt’s voles and model species were identified through reciprocal best hits (RBHs), where two proteins from different species mutually identify each other as top matches across proteomes [25]. Sequence alignment was performed using the Needleman–Wunsch global alignment algorithm implemented in the Biostrings R package with default parameters. The resulting alignments were used to map the sequence positions of phospho-sites detected in Brandt’s voles to the corresponding positions in the protein sequences of the model species.

The phospho-site functional scores of Brandt’s voles were calculated using the funscoR R package (https://github.com/evocellnet/funscoR, accessed on 1 February 2025), with scores > 0.5 indicating five- to tenfold increased impact on molecular interactions or phenotypes [26]. Kinase activity was predicted through a kinase-substrate enrichment algorithm (KSEA) [27] using phosphorylation ratios between hypoxia and normoxia. Known kinase-substrate site relationships were downloaded from PhosphoSitePlus (last modified 23 May 2023) [28]. Kinases with *p* < 0.05 and ≥2 measured substrates were considered significantly altered.

### 2.11. Weighted Gene Correlation Network Analysis (WGCNA)

Co-expression networks for DE proteins and phospho-sites were carried out with the WGCNA R package [29]. The analysis process mainly included the following steps: (1) defining Pearson correlation matrix (with direction, i.e., for building signed co-expression network); (2) converting the correlation matrix into an adjacency matrix by selecting the weighting coefficient; (3) merging modules with similar expression patterns by assessing the representative pattern of each calculation module and the quantitative similarity among them; (4) calculating a consensus trend for each module based on the eigengene. Proteins and phospho-sites were assigned to the most relevant cluster using a Pearson correlation cutoff of 0.7.

To facilitate further analysis, we categorized the clusters into four groups based on the method described by Qi et al. [30]: (1) Up group: Proteins or phospho-sites whose expression levels increase with altitude. (2) High group: Proteins or phospho-sites whose expression levels at 3000 m, 5000 m, or 7000 m are higher than those at 100 m. (3) Down group: Proteins or phospho-sites whose expression levels decrease with altitude. (4) Low group: Proteins or phospho-sites whose expression levels at 3000 m, 5000 m, or 7000 m are lower than those at 100 m.

### 2.12. Western Blotting

Brain lysates were denatured and separated by SDS-PAGE and transferred to PVDF membranes. After blocking with 5% non-fat milk (1 h, RT), membranes were incubated with the specific primary antibodies overnight at 4 °C: SSRP1 (PTMab, PTM-6446, 1:1000, Hangzhou, China), Phospho-Camk2a-T286 (Abclonal, AP0255, 1:1000, Wuhan, China), CAMK2A (PTMab, PTM-6299, 1:1000) and β-actin (PTMab, PTM-5028, 1:2000). Following secondary antibody incubation (2 h, RT), proteins were detected using an ECL kit (YESEN, 36208ES60, Shanghai, China) and captured on a ChemiDoc Imaging System (Bio-Rad, Hercules, CA, USA). Band intensities were quantified using ImageJ 1.54 and normalized to β-actin.

### 2.13. Statistical Analysis

Data were presented in the format of mean  ±  SEM, while statistical analysis was performed using GraphPad Prism 9 software. Statistically significant differences between hypoxic and normoxic groups were assessed using Student’s *t*-test, and *p* value < 0.05 was deemed significant for all analyses conducted.

## 3. Results

### 3.1. Quantitative Proteome and Phosphoproteome Profiling

**Figure 1:**
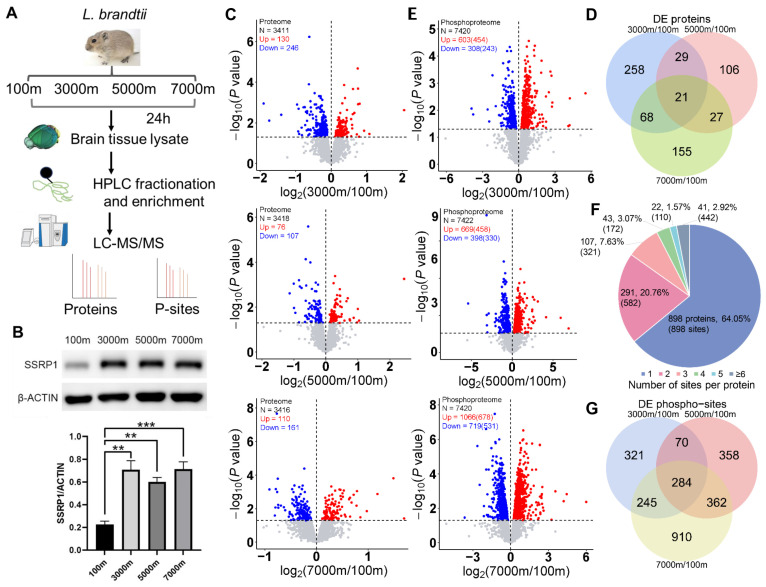
Proteomic and phosphoproteomic profiling of Brandt’s vole brain tissue under high-altitude hypoxia. (**A**) Schematic overview of the experimental design. (**B**) Western blot analysis of selected protein (*n* = 3/group, mean ± SEM). (**C**) Volcano plots showing protein expression across altitude comparisons. (**D**) Venn diagram illustrating the overlap of DE proteins. (**E**) Volcano plots of phospho-site expression changes. (**F**) Distribution of number of phospho-sites per protein. Percentages reflect the proportion of proteins within each category. Parentheses indicate the aggregate phospho-sites detected per group. (**G**) Venn diagram showing the overlap of DE phospho-sites. ** *p* < 0.01; *** *p* < 0.001.

In order to systematically characterize hypoxia-induced molecular changes in the brain tissue of Brandt’s voles, we conducted quantitative profiling of both the proteome and phosphoproteome (Figure 1A). A total of 3990 proteins (Table S1) and 9125 phospho-sites (Table S2) were quantitatively profiled. To validate our omics data, we performed Western blotting of SSRP1, a component of the FACT complex that regulates stress-induced gene transcription upon cellular stimulation [31]. The experimental results showed consistent trends with the omics data, confirming the reliability of our profiling approach (Figure 1B). Proteomic analysis revealed a limited number of significantly altered proteins in the comparisons between 3000 m and 100 m (376 proteins), 5000 m and 100 m (183 proteins), and 7000 m and 100 m (271 proteins) (Figure 1C). Overall, 242 proteins were upregulated and 422 were downregulated, with only 21 DE proteins common to all comparison groups (Figure 1D). Enrichment analysis of DE proteins in each comparison group demonstrated that the cellular impact of hypoxia progressively shifted from nervous system, vascular, and metabolic regulation at lower altitudes to more pronounced apoptosis, stress responses, and immune regulation at higher altitudes (Table S3). In contrast, phosphoproteomic analysis revealed a more pronounced response, with significantly altered phospho-sites increasing with altitude: 911 sites (3000 m vs. 100 m), 1067 sites (5000 m vs. 100 m), and 1785 sites (7000 m vs. 100 m) (Figure 1E). We identified 1523 upregulated and 1043 downregulated phospho-sites, with approximately 36% of proteins containing multiple modified sites (Figure 1F and Table S4). Among these, 284 DE phospho-sites were observed across all comparisons (Figure 1G). We found that the pathways enriched in phospho-proteins were similar across the three groups, including nervous system and synaptic function, cytoskeletal organization, and metabolic regulation (Table S3). While changes in relative protein levels may facilitate long-term adaptive adjustments, post-translational modifications can rapidly transduce hypoxia responses through direct effects on protein stability [32].

**Figure 2:**
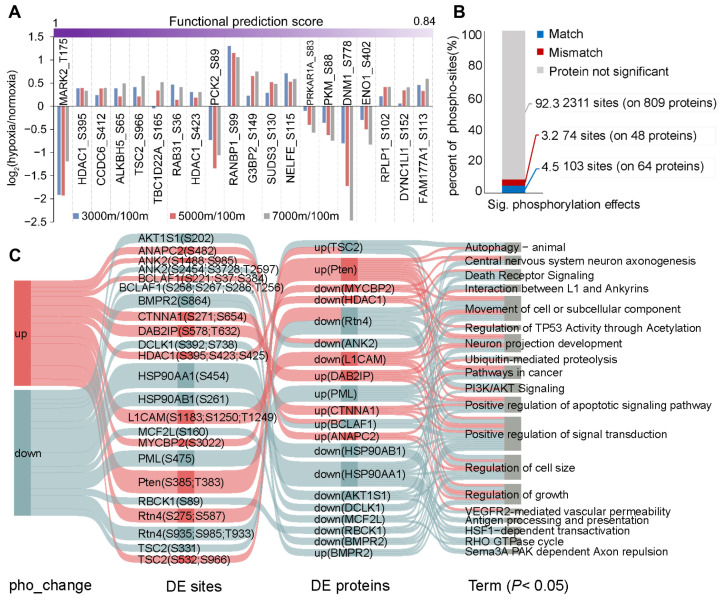
Functional characterization of DE phospho-sites. (**A**) Top 20 phospho-sites ranked by functional prediction scores. (**B**) Proportion of significantly altered phospho-sites showing match or mismatch changes in protein abundance, with gray indicating non-significant changes of proteins. (**C**) Pathways enriched for proteins exhibiting changes in both expression and phosphorylation levels. The network architecture comprises four distinct layers: phosphorylation clusters (far left), individual sites (second layer), protein-level patterns (third layer), and enriched pathways (final layer). Pathway information was compiled from three established databases: GO, KEGG, and Reactome.

However, the functional significance of many significantly altered phospho-sites remains uncharacterized in the literature. Therefore, we employed a machine learning-based approach to perform scoring [26], identifying 391 phospho-sites with known or high predicted functional scores (Figure 2A and Table S5). For example, phosphorylation at T208 activates MARK2, a regulator of cell polarity and microtubule dynamics, leading to microtubule fragmentation and cell death [33]. Interestingly, reduced phosphorylation of MARK2 at T175 (the homologous site to T208 in human) appears neuroprotective. These findings suggest that phosphorylation-mediated regulation in Brandt’s voles may provide potential therapeutic targets for preventing and treating acute mountain sickness and other hypoxia-related neurological disorders. In addition, we assessed the contribution of protein abundance to phospho-level variations. Most significantly altered phospho-sites showed no corresponding significant changes in protein abundance (Figure 2B). Among 112 proteins exhibiting changes at both levels, enrichment analysis identified PTEN-mediated PI3K/AKT signaling as a potential mechanism promoting cell survival in Brandt’s voles (Figure 2C).

Additionally, integrating previous transcriptomic studies revealed that immune response, metabolic, and vascular regulation pathways were identified across all three omics layers [14,15]. DE mRNAs and proteins were predominantly linked to oxidative stress, whereas phospho-proteins were more involved in signal transduction and cytoskeletal regulation. However, the overlap of DE proteins was limited, potentially due to lag between transcription and translation (Figure S1). These findings provide a multidimensional view of the molecular mechanisms underlying hypoxia adaptation in Brandt’s voles.

### 3.2. Expression Dynamics and Functional Enrichment Analysis in DE Proteins

**Figure 3:**
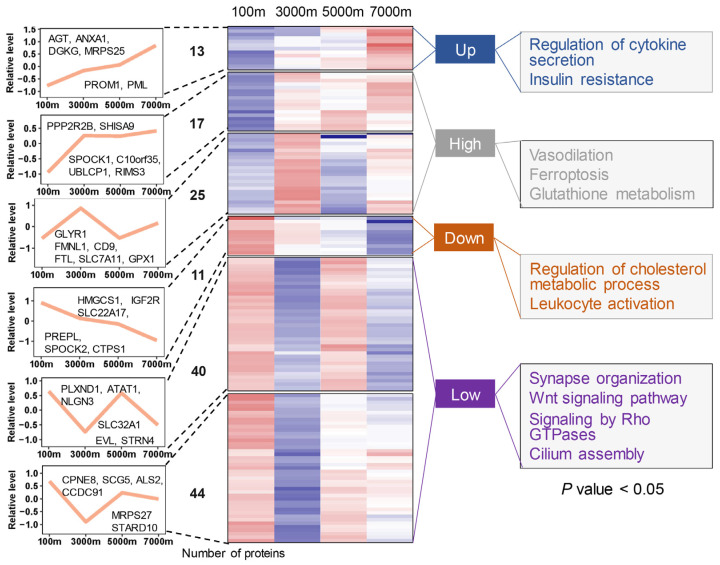
Proteome profiling of Brandt’s vole brain tissue across altitudinal gradients reveals co-expression clusters and significant enrichment of functional annotations. The number of DE proteins for each cluster is listed between dashed lines. In the heatmap, the color gradient from blue to red represents low to high expression levels, respectively.

To explore the protein dynamics of Brandt’s voles under simulated high-altitude hypoxia, these DE proteins were divided into six co-expression clusters by WGCNA (Figure 3). For the sake of convenient analysis, we subsequently categorized these clusters into four groups: up, high, down, and low [30]. The up group, comprising AGT, ANXA1, and DGKG, showed increasing expression with altitude and was enriched in regulation of cytokine secretion, insulin resistance, and so on (Table S6), suggesting enhanced inflammatory modulation and metabolic adaptation. The high group, containing PPP2R2B, SLC7A11, and GPX1, exhibited irregular fluctuations and was correlated with vasodilation, ferroptosis, and glutathione metabolism, indicating improved oxygen delivery and oxidative stress resistance. The down group showed a monotonic downregulation pattern and contained HMGCS1, IGF2R and SLC22A17. Pathway analysis revealed enrichment in regulation of the cholesterol metabolic process and leukocyte activation, likely reflecting energy conservation strategies. Interestingly, Tibetan pigs exhibit lower IGF2R expression compared with their lowland counterparts, and IGF2R inhibition alleviates hypoxia-induced cell death [33]. The low group, containing the majority of DE proteins (e.g., PLXND1, ATAT1), showed non-monotonic downregulation and was enriched in synapse organization, Wnt signaling pathway, signaling by Rho GTPases, and so on. Research has found that inhibition of PLXND1-mediated Rho GTPases signaling promotes vascular network recovery following cerebral ischemia [34]. Importantly, many identified proteins, such as IGF2R, have been reported as candidate genes in other species (Figure S2), suggesting the adaptive mechanisms to hypoxia in Brandt’s voles.

### 3.3. Expression Dynamics of Phosphorylation Events and Enriched Pathways in Phospho-Proteins

**Figure 4:**
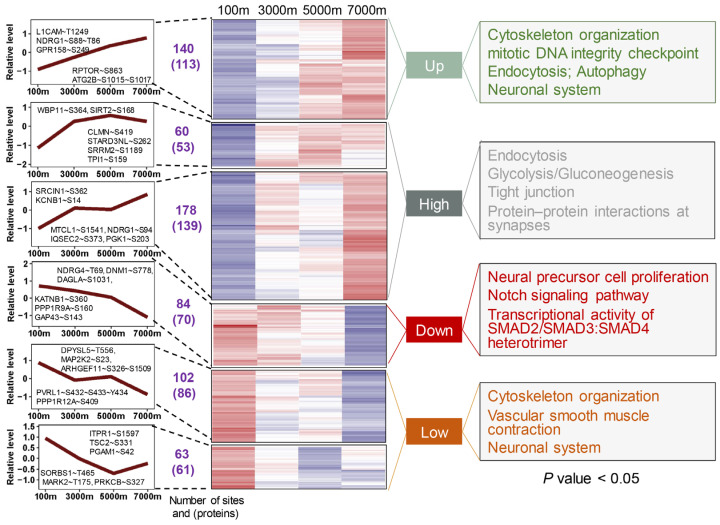
Phosphoproteome profiling of Brandt’s vole brain tissue across altitudinal gradients identifies co-expression clusters and significant enrichment of functional annotations. The number of DE phospho-sites and phospho-proteins for each cluster is listed between dashed lines. In the heatmap, the color gradient from blue to red represents low to high expression levels, respectively.

The WGCNA of DE phospho-sites yielded six clusters, which could be divided into four groups (Figure 4). Compared with proteome, the dynamic changes of phosphoproteome seemed to be more regular. The monotonically up group was mainly enriched in cytoskeleton organization, endocytosis, and autophagy (Table S6). Hypoxia-induced autophagy exhibits dual roles in neuroprotection and damage, with phosphorylation of ATG2B and RPTOR-S863 potentially regulating the autophagic network [35,36]. The high group, representing the largest cluster of phospho-sites, was related to endocytosis, tight junction, and glycolysis/gluconeogenesis. The down group displayed monotonic decreases in phosphorylation levels and was enriched in neural precursor cell proliferation and the Notch signaling pathway. The low group showed non-monotonic decreases with altitude and was associated with cytoskeleton organization, vascular smooth muscle contraction, and the neuronal system. Although certain pathways, such as cytoskeleton organization, endocytosis, and those of the nervous system, appeared in multiple groups (Table S6), most of the phospho-proteins within these pathways were distinct (Figure S3), reflecting complex spatiotemporal regulation. The significant enrichment of cytoskeletal phospho-proteins likely reflects the cytoskeleton’s dynamic nature and phosphorylation-dependent regulation [37]. Beyond providing structural support, the cytoskeleton plays crucial roles in cell migration, stress responses, and endocytosis [38]. It has been reported that oxygen-glucose deprivation disrupts cytoskeletal organization and induces cell death in rat hippocampal neurons [39]. These findings demonstrate that phosphorylation-mediated regulation of key pathways represents a critical adaptive mechanism for maintaining neuronal function and survival under hypoxic conditions in Brandt’s voles.

### 3.4. The Pivotal Kinases Involved in Hypoxic Regulation

**Figure 5:**
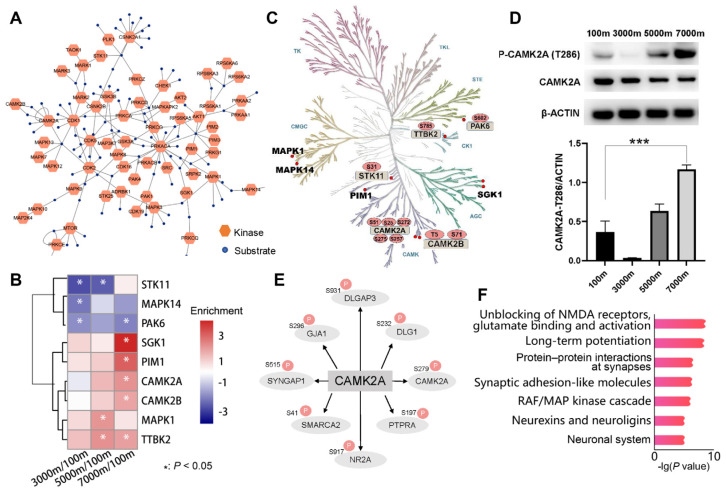
Key regulatory kinases involved in hypoxic response in Brandt’s vole brain tissue. (**A**) The network constructed from 83 kinases and their 206 substrates from Brandt’s vole. (**B**) Nine kinases exhibiting significant change in activity in at least one comparison. (**C**) Canonical phospho-sites on kinases mapped onto the kinome phylogenetic tree. (**D**) Western blot of selected kinase (*n* = 3/group, mean ± SEM). (**E**) CAMK2A substrates. (**F**) Significantly enriched pathways among CAMK2A substrates identified through functional enrichment analysis. *** *p* < 0.001.

Protein kinases serve as crucial upstream regulators of phosphorylation cascades, and their activity status can be inferred from the phosphorylation levels of their substrates. We first constructed a regulatory network comprising 83 kinases and 206 substrates in Brandt’s voles (Figure 5A). To enhance result reliability, only 30 kinases detected across all comparison groups were retained, among which 9 exhibited significant activity alterations (Figure 5B and Table S7). STK11, MAPK14, and PAK6 showed decreased activity in at least one treatment group, while SGK1, PIM1, CAMK2A, CAMK2B, MAPK1, and TTBK2 exhibited increased activity at 5000 m or 7000 m. Five kinases were identified to directly affect phosphorylation regulation (Figure 5C). A literature review revealed seven kinases with established hypoxia-related functions. For instance, MAPK1 exhibits cell-type-specific effects in neonatal hypoxic-ischemic brain injury, promoting neuronal damage while exerting neuroprotection in astrocytes [40]. In addition, our analysis identified four CAMK family members, suggesting their potential role in hypoxic response (Figure 5C). Remarkably, CAMK2A activity showed an altitude-dependent pattern: slight downregulation at 3000 m, followed by progressive increases at higher altitudes. Immunoblot experiments validated these activity changes (Figure 5D). CAMK2A activity was calculated based on eight known substrates (DLGAP3, DLG1, CAMK2A, PTPRA, GRIN2A, SMARCA2, SYNGAP1, and GJA1) (Figure 5E). Enrichment analysis of these substrates highlighted the crucial role of phosphorylation in maintaining synaptic function during hypoxic adaptation (Figure 5F).

## 4. Discussion

**Figure 6:**
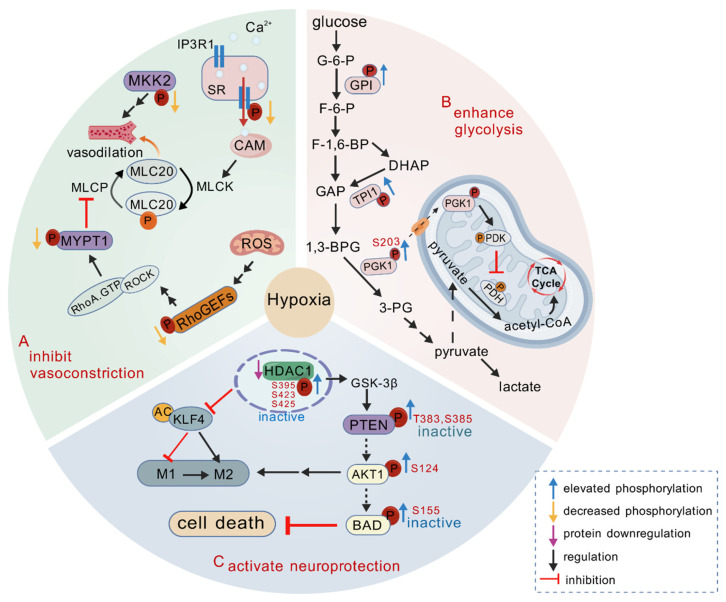
Regulatory mechanisms of Brandt’s vole brain tissue in response to hypoxia. Under hypoxic conditions, Brandt’s voles may cope with hypoxia through three key mechanisms: (**A**) inhibition of vascular smooth muscle contraction to increase blood flow, (**B**) enhancing glycolysis to provide energy, and (**C**) activation of HDAC1 and the PI3K-Akt signaling pathway to reduce cell death. The schematic was created using the Generic Diagramming Platform (GDP) (https://biogdp.com/, accessed on 1 December 2024).

In this study, we employed quantitative mass spectrometry to systematically characterize proteomic and phosphoproteomic dynamics in Brandt’s vole brain tissue during high-altitude hypoxia. We observed that, although protein abundance changes were relatively modest with increasing altitude, the cellular response to hypoxia gradually shifted from neural, vascular, and metabolic regulation to more pronounced apoptosis, stress responses, and immune modulation. Most co-expressed proteins exhibited non-monotonic responses, consistent with previous high-altitude adaptation studies [30,41,42]. This pattern may be attributed to their blunt hypoxia tolerance shaped by long-term natural selection, which minimizes excessive molecular responses that could be toxic to cells [30], or intricate complex regulatory mechanisms involving transcription factors, enhancers, and repressors, as well as feedback loops [43,44]. In contrast, phosphorylation exhibited substantial changes in response to acute hypoxia, with similar enriched pathways across comparison groups, including synaptic function, cytoskeletal organization, and metabolic regulation. The predominantly monotonic response patterns of co-expressed phospho-sites suggest that phosphorylation plays a rapid, specific, and central regulatory role in hypoxic adaptation. Additionally, we identified several key kinases potentially involved in hypoxia regulation. Collectively, both DE proteins and DE phospho-proteins were implicated in vascular function regulation, metabolic reprogramming, synaptic plasticity, and apoptosis. The rapid responsiveness of phosphorylation, coupled with the long-term regulation of gene expression, synergistically maintains cellular homeostasis under hypoxic conditions (Figure 6).

### 4.1. Regulation of Cerebral Blood Flow Through Vasoconstriction Inhibition

Arteries are exquisitely sensitive to tissue oxygen fluctuations. Hypoxia-induced mitochondrial reactive oxygen species (ROSs) and cellular Ca^2+^ imbalances trigger vasomotor responses to optimize oxygen and nutrient delivery [45]. In Brandt’s voles, hypoxia exposure decreased phosphorylation of key vascular regulators: MKK2-S23, MYPT1-S409, PDZ-RhoGEF at S326 and S1509, IP3R1-S1597, and PRKCB-S327, potentially aiding in the inhibition of vascular smooth muscle contraction (Figure 6A). It has been reported that ROSs induced by hypoxia or vascular diseases can pre-activate RhoA through RhoGEFs, thereby mediating an increase in Rho kinase activity. This, in turn, phosphorylates MYPT1, which in its phosphorylated state inhibits the activity of MLCP, leading to the activation of MLC20 and subsequently triggering vasoconstriction [46,47]. Furthermore, the phosphorylation of IP3R1 triggers the release of Ca^2+^ from the sarcoplasmic reticulum into the cytosol, followed by sequential activation of calmodulin proteins, MLCK, and MLC20, ultimately inducing smooth muscle contraction [48]. Supporting evidence shows that MKK2 inhibition alleviates subarachnoid hemorrhage-induced vasoconstriction [49], and hypoxia-induced pulmonary vasoconstriction in rats can be ameliorated through PRKCB inhibition [50]. Mechanistically, reduced phosphorylation of these regulators plays a pivotal role in suppressing vasoconstriction, thereby facilitating the preferential redistribution of cerebral blood flow to brain regions with higher oxygen demands in Brandt’s voles.

Additionally, tissues can enhance oxygen supply efficiency through mechanisms such as vascular remodeling. Our analysis revealed that Brandt’s voles dynamically regulate VEGFR2-mediated vascular permeability and cerebrovascular morphogenesis via the expression of PLXND1, GPR56, CDH13, RTN4, AAMP, and BECN1 (Table S3). Previous transcriptomic studies showed that under hypoxic conditions, Brandt’s voles regulate angiogenesis in the brain through Angpt2, EDN1, VEGFA, THBS1, and MMP2 [14]; in skeletal muscle, CYP- and COL-family genes promote erythrocyte oxygen transport and angiogenesis [17]; and in the lungs, angiogenesis is inhibited by changes in Angpt2, Adamts8, and Thbs1, while oxygen transport is enhanced through upregulation of erythropoiesis- and hemoglobin-synthesis-related genes [16]. This coordinated and tissue-specific response highlights the sophisticated molecular mechanisms underlying hypoxia adaptation in Brandt’s voles, providing valuable insights into potential therapeutic targets for hypoxia-related conditions.

### 4.2. Regulation of Cellular Energetics

Brain tissue lacks the ability to store energy, thus reducing energy consumption and upregulating anaerobic metabolism are crucial for hypoxic tolerance [51]. For instance, the western painted turtle is a recognized vertebrate tolerant to hypoxia, during which its overall protein synthesis rate in the brain decreases to undetectable levels [52]. Our proteomic analysis found that there was a significantly higher number of downregulated proteins compared with upregulated proteins in Brandt’s voles, suggesting a reduction in protein synthesis as an energy-saving mechanism.

Phosphoproteomic analysis of Brandt’s voles identified significant enrichment of the glycolysis/gluconeogenesis pathway in the high group. The phosphorylation levels of GPI-S43, TPI1-S159, and PGK1-S203 were upregulated under different hypoxic conditions, indicating glycolysis activation as a universal adaptive mechanism in Brandt’s voles, regardless of its severity. TPI1 is a quintessential glycolytic enzyme positioned at a metabolic crossroads. Recent two reports have indicated that the phosphorylation of TPI at either S21 or S58 in lung cancer tissues may endow TPI with a more dynamic and active conformation, facilitating the conversion of DHAP to GAP and thereby significantly enhancing glycolytic flux [53,54]. In this study, we propose that the increased phosphorylation level of TPI1-S159 in Brandt’s voles may enhance the glycolytic process. PGK1 catalyzes one of the two ATP-generating reactions in the glycolytic pathway. Previous studies have demonstrated in cancer cells that PGK1 phosphorylation at S203, mediated by ERK1/2 under hypoxia, promotes mitochondrial translocation, where it phosphorylates PDK1 and PDH, reducing mitochondrial pyruvate utilization while suppressing ROSs and enhancing cytosolic pyruvate and lactate production [55,56]. Nevertheless, lactate accumulation can trigger metabolic disturbances and cellular injury. Our recent findings revealed that Brandt’s voles employ PPRC1-regulated gluconeogenesis in the liver, maintaining metabolic homeostasis during hypoxia [57].

### 4.3. Activating Neuroprotective Mechanisms

Despite enhanced cerebral blood flow and ATP production, high-altitude hypoxia-induced redox imbalance and inflammatory responses may still lead to neuronal damage and cerebral edema [51]. Our proteomic profiling demonstrated that the expression levels of SLC7A11 and GPX1 were significantly elevated at 3000 m, likely representing an adaptive strategy in Brandt’s voles to counteract oxidative stress under mild hypoxia. Notably, dexamethasone (commonly used for the prevention and treatment of acute mountain sickness) and *Ligusticum* Chuanxiong have been reported to exert neuroprotective effects by reducing inflammatory factors and upregulating SLC7A11 and GPX [58]. Additionally, previous research has shown that Brandt’s voles can enhance cerebral antioxidant defense through increased ascorbic acid levels [11]. Furthermore, ANXA1, an anti-inflammatory mediator, showed significant elevation at 7000 m, suggesting its potential role as a protective mechanism against severe hypoxia-induced brain injury in Brandt’s voles. Supporting evidence indicates that exogenous ANXA1 administration alleviates blood-brain barrier disruption and inflammation in brain-injured mice [59].

Hypoxia-induced glutamate stimulates the NMDA receptor, triggering Ca^2+^ influx that induces CaMK2 autophosphorylation at Thr286 and activates its kinase activity. Kinase activity profiling revealed a progressive upregulation of CaMK2A activity in Brandt’s voles with increasing altitude, accompanied by corresponding elevation in phosphorylation levels of its substrate DLG1-S232. Notably, NR2A-S917 phosphorylation (the homologous site to NR2A-S1459 in humans) showed significant upregulation exclusively at 7000 m. Mechanistically, DLG1-S232 phosphorylation disrupts the DLG1/NR2A complex, facilitating NR2A insertion into the postsynaptic membrane [60], while NR2A-S1459 phosphorylation regulates NMDA receptor recycling and gating, potentially influencing synaptic plasticity [61]. The salidroside derivative SHPL-49 has been shown to mitigate glutamate excitotoxicity in acute ischemic stroke through activation of the NR2A-CAMK2A-Akt/CREB signaling pathway [62]. These findings suggest that Brandt’s voles may maintain neural function through the CaMK2A-T286/DLG1-S232/NR2A-S917 axis with increasing altitude.

The PTEN-mediated PI3K/Akt pathway, crucial for neuroprotection and neuronal death [63], was activated in hypoxic Brandt’s voles. PTEN carboxyl-terminal tail contains multiple phospho-sites (Ser/Thr residues 380–385), and their phosphorylation inhibits phosphatase activity by inducing a stable, closed conformation [64]. Phosphorylated PTEN prevents the degradation of PIP3, thereby facilitating AKT activation. Our results showed that the phosphorylation levels of PTEN at T383 and S385, as well as AKT at S124, increased progressively with altitude and were significantly elevated at 7000 m in Brandt’s voles, likely contributing to the activation of pro-survival pathways. Although research on AKT-S124 phosphorylation remains limited, existing studies suggest that elevated AKT-S124 levels may reduce hippocampal neuronal death [65] or promote tumorigenesis in gliomas [66]. BAD, a well-known pro-apoptotic protein, can be inactivated through phosphorylation at one or more sites. Interestingly, BAD-S155 was significantly upregulated in Brandt’s voles at 3000 m, potentially blocking its pro-apoptotic activity. However, no direct evidence currently links BAD-S155 to AKT as a downstream target. Under severe hypoxia, activated AKT may regulate cellular functions by phosphorylating other downstream effectors. Additionally, we observed that hypoxia exposure induced upregulated phosphorylation of HDAC1 at S395, S423 (the homologous site to S421 in humans), and S425 (the homologous site to S423) in Brandt’s voles, while its protein levels were downregulated (Figure 2B). It has been demonstrated that genetic ablation of HDAC1 or promotion of its phosphorylation at S421 and S423 in the mouse hippocampus confers neuroprotection [67]. Inhibition of HDAC expression or promotion of HDAC phosphorylation may promote the polarization of microglia from the detrimental M1 phenotype to the beneficial M2 phenotype, either through activation of the GSK3B/PTEN/Akt axis [68] or inhibition of KLF4 deacetylation [69], ultimately providing neuroprotection by mitigating neuroinflammation. Although further experiments are necessary to elucidate these mechanisms, the above findings underscore the positive role of phosphorylation in the neuroprotection of Brandt’s voles.

## 5. Conclusions

This study provides the first comprehensive characterization of proteomic and phosphoproteomic dynamics in Brandt’s vole brain tissue during hypoxic adaptation. Through integrative multi-omics analysis, our findings revealed three major adaptive strategies in Brandt’s voles: (1) inhibition of vascular smooth muscle contraction to increase blood supply to the brain tissue; (2) metabolic rewiring through glycolytic activation to maintain energy homeostasis; and (3) activation a series of neuroprotective mechanisms, including mitigation of oxidative stress and inflammatory responses, and PI3K/AKT signaling regulation (Figure 6). These comprehensive datasets provide valuable resources for hypoxia adaptation research and potential therapeutic insights for altitude-related pathologies. However, this study has several limitations. First, the sample size was limited by both economic constraints and ethical considerations regarding animal use. Second, the study focused on a single time point, leaving the mechanisms underlying long-term chronic hypoxia and intermittent hypoxia unexplored. Additionally, experimental constraints restricted mechanistic validation of proposed molecular pathways. Furthermore, cellular heterogeneity poses a challenge in attributing observed changes to specific cell types. Future studies incorporating single-cell sequencing technologies could precisely identify cell-type-specific regulatory mechanisms, while functional validation experiments would further elucidate the molecular basis of hypoxic adaptation.

## Data Availability

The mass spectrometry proteomic and phosphoproteomic data have been deposited in the ProteomeXchange Consortium via the iProX partner repository [70] with the dataset identifier PXD056880.

## Supplementary Materials

The following supporting information can be downloaded at: https://www.mdpi.com/article/10.3390/cells14070527/s1, Figure S1: Overlap of the differentially expressed (DE) mRNAs, DE proteins and DE phosphoproteins; Figure S2: The DE proteins have been identified as candidate genes in other hypoxia-tolerant species; Figure S3: Phosphoprotein overlap analysis; Table S1: Proteomics data; Table S2: Phosphoproteomics data; Table S3: Enriched pathways of DE proteins and DE phospho-proteins; Table S4: Enumeration of phospho-sites for individual proteins; Table S5: Functional scoring of DE phospho-sites; Table S6: Enriched pathways in clustered proteins and phosphoproteins; Table S7: Kinase activity analysis.
