# Supplementary material for: Integrative Proteomic and Phosphoproteomic Profiling Reveals Molecular Mechanisms of Hypoxic Adaptation in Brandt’s Voles (Lasiopodomys brandtii) Brain Tissue
Source: Cells. 2025 Apr 1;14(7):527. doi: 10.3390/cells14070527 (PMC11988865; doi:10.3390/cells14070527)
Supplement: Supplementary file 1 [file cells-14-00527-s001.zip › Supplemental Figures.pdf]

Supplemental Figures

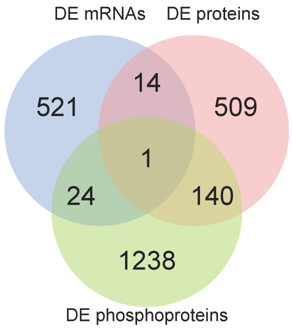

Figure S1. Overlap of the differentially expressed (DE) mRNAs, DE proteins and DE phosphoproteins. The differentially expressed mRNAs are derived from the previously published transcriptomic data (Dong et al., Frontiers in zoology 17 (2020) 9, and BMC Genomics 19(1) (2018) 901).

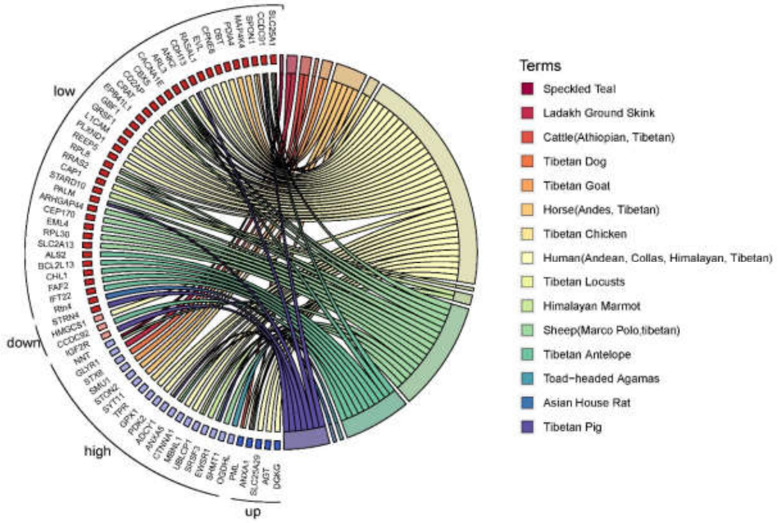

Figure S2. The DE proteins have been identified as candidate genes in other hypoxia-tolerant species.

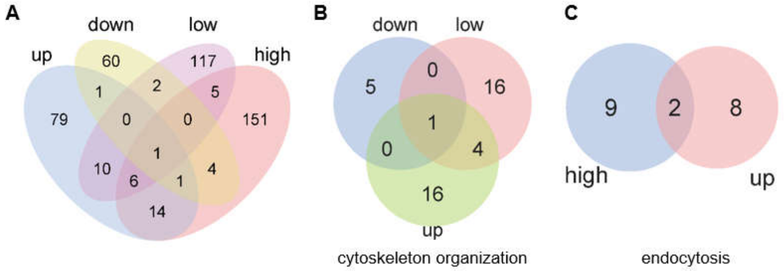

Figure S3. Phosphoprotein overlap analysis. (A) Cluster commonality. (B) Cytoskeleton organization Commonality. (C) Endocytosis commonality.
